# Bile is a reliable and valuable source to study cfDNA in biliary tract cancers

**DOI:** 10.3389/fonc.2022.961939

**Published:** 2022-08-26

**Authors:** Zhanghui Li, Yelei Liu, Junhui Fu, Joseph Mugaanyi, Junrong Yan, Caide Lu, Jing Huang

**Affiliations:** ^1^ Department of Hepato-Pancreato-Biliary Surgery, Ningbo Medical Center Lihuili Hospital, The Affiliated Hospital of Ningbo University, Ningbo, China; ^2^ Medical Department, Nanjing Geneseeq Technology Inc., Nanjing, China

**Keywords:** biliary tract carcinoma, bile, next-generation sequencing, chromosomal instability, liquid biopsy

## Abstract

**Objective:**

The aim of this study is to determine the clinical efficacy of bile-derived liquid biopsy compared with plasma and tumor tissue biopsy in patients with biliary tract carcinoma (BTC).

**Methods:**

A total of 13 patients with BTC were enrolled in this cohort. Tumor tissue, bile, and plasma samples were obtained and analyzed using next-generation sequencing for genomic profiling.

**Results:**

Bile and plasma samples were collected from all 13 patients, and 11 patients also had matched tumor tissues available. The cell-free DNA (cfDNA) concentration was significantly higher in the bile supernatant than in plasma (median: 1918 vs. 63.1 ng/ml, p = 0.0017). The bile supernatant and pellet had a significantly higher mean mutation allele frequency (MF) than plasma (median: 3.84% vs. 4.22% vs. 0.16%; p < 0.001). Genomic alterations were predominantly missense. Both bile supernatant and pellet had significantly more genomic alterations than plasma (average: 9.3 vs. 7.2 vs. 2.3 alterations per sample; p < 0.01). Among the top 10 most frequent genomic alterations, the consistency between bile supernatant and tumor tissue was 90.00% (18/20), that between bile pellet and tumor tissue was 85.00% (17/20), and that between the plasma and tissue was only 35.00% (7/20). MAF of both bile supernatant and pellet was positively correlated with that in tissue samples (ρ < 0.0001, spearman r = 0.777, and ρ < 0.0001, spearman r = 0.787, respectively), but no significant correlation with tissue was found in the plasma (ρ = 0.966, spearman r = 0.008). Furthermore, additional genomic alterations could be detected in bile supernatant and pellet than in tissue. Potential targets for targeted therapy were identified in bile supernatant and pellet. Regarding copy number variation (CNV) and chromosomal instability (CIN) detection, four additional CNVs from two patients were detected in the bile supernatant that was not detected in tissues (i.e., amplification of *TERC*, *IL7R*, *RICTOR*, and *TERT*). CIN was significantly higher in tumor tissue than in plasma. The CIN of the bile was also significantly higher than that of plasma. There was no significant difference in CIN between the tissue and the bile supernatant.

**Conclusion:**

The consistency of all genomic alterations and tumor tissue-determined genomic alteration in the bile supernatant/pellet was significantly higher than in plasma. Bile supernatants/pellets are better for genetic sequencing and may also have potential clinical value to guide targeted therapy and evaluate prognosis. Bile cfDNA may be a feasible substitute for tumor tissue in the genetic testing of patients with BTC.

## Introduction

Biliary tract carcinoma (BTC) includes intrahepatic cholangiocarcinoma, hilar cholangiocarcinoma, distal cholangiocarcinoma, and gallbladder carcinoma. In the past two decades, the global incidence rate of BTC has increased yearly (1). Because of a lack of typical/specific clinical manifestations in the early stages of BTC, most patients are ineligible for surgical resection at the time of diagnosis (2). Carbohydrate antigen 199 and carcinoembryonic antigen are the most commonly used tumor biomarkers for diagnosing BTC. However, their specificity is not high enough, with only 71.7% and 11.5%, respectively (3). Given the anatomical location of the biliary tract and its proximity to other vital and visceral structures, the screening, diagnosis, and treatment of BTC are extremely difficult. Endoscopic ultrasound-guided fine-needle aspiration and endoscopic cholangiopancreatography combined with brush biopsy are increasingly being used to diagnose BTC; however, their sensitivity is not very high, and they may present some severe complications (4). Over the past decade, with the continuous innovation and developments in medical technology, from polymerase chain reaction (PCR) to next-generation sequencing (NGS), research on circulating tumor DNA (ctDNA) has increased. However, most studies have focused on using genetic alterations to predict prognosis or guide treatment decisions (5). Bile liquid biopsy, which is in direct contact with the tumor tissue, may more accurately reflect the tumor tissue’s genetic alterations status. As such, we conducted a retrospective study to investigate this theory. In this study, we collected bile and plasma from 13 patients with BTC; 11 patients also had matched tumor tissues. Bile samples were separated into supernatant and pellet for independent extraction of cell-free DNA (Bile-cfDNA) and sediment DNA (Bile-sDNA). All specimens were subject to molecular profiling using target NGS of 425 cancer-relevant genes. The aim was to evaluate the concordance of genomic profiles between tumor tissue and the other different sample types and determine whether bile-derived cfDNA detection could be a possible replacement for tumor tissue genetic testing for clinical molecular diagnoses.

## Materials and methods

Between June 2021 and January 2022, a total of 13 patients with BTC were enrolled at Ningbo Medical Center Li Huili Hospital. The study was approved by the ethics committee of Ningbo Medical Center Li Huili Hospital, and all patients provided written informed consent (ethics approval number: KY2021JP240). All samples were tested at a clinical genomic testing center (Nanjing Geneseeq Technology Inc.).

For each patient, 8–10 ml of peripheral blood was collected. Plasma and white blood cells (WBCs) were separated using centrifugation at 1,800 rpm for 10 min within 2 h of collection. The isolated plasma was used for the extraction of cfDNA, and the WBCs were used for the extraction of genomic DNA as a negative control. Formalin-fixed tumor tissue samples were obtained from 11 of the 13 patients. An experienced pathologist determined the tumor cell content of each sample. A 3- to 6-ml aliquot of bile was centrifuged at 2,500 rpm for 15 min to separate the supernatant from the pellet. The supernatant was isolated for cfDNA extraction, and the genomic DNA was extracted from the pellet and used as sDNA. For cytological assessment of tumor cells in bile, cell smears were prepared from the pellet of another aliquot of bile and stained with hematoxylin and eosin. A pathologist examined the contents of each smear under magnification of ×400.

cfDNA from plasma and bile supernatant was purified using the Circulating Nucleic Acid Kit (Qiagen, Hilden, Germany) following the kit manufacturer’s protocol. Genomic DNA was extracted from the bile pellets and leukocytes using the DNeasy Blood & Tissue Kit (Qiagen). Formalin-fixed, paraffin-embedded (FFPE) genomic DNA was purified using the QIAamp DNA FFPE Tissue Kit (Qiagen). Genomic DNA was characterized using a Nanodrop2000 (Thermo Fisher Scientific, Waltham, MA), and cfDNA fragment distribution was analyzed on a Bioanalyzer 2100 using the High Sensitivity DNA Kit (Agilent Technologies, Santa Clara, CA). All DNA was quantified using the dsDNA HS detection kit on a Qubit 3.0 Fluorometer (Life Technologies, Carlsbad, CA).

### Library preparation and sequencing

cfDNA or fragmented genomic DNA (300~350 bp with Covaris M220 instrument) underwent sequencing library preparation using the KAPA Hyper Prep kit (KAPA Biosystems). In brief, DNA was experienced with end-repairing, A-tailing, adapter ligation, and size selection using Agencourt AMPure XP beads (Beckman Coulter) and then was amplified by PCR and purified.

Indexed DNA libraries were pooled up to 2 µg, together with Human cot-1 DNA (Life Technologies) and xGen Universal blocking oligos (Integrated DNA Technologies) as blocking reagents. A customized xGen lockdown probe panel (Integrated DNA Technologies) covering 425 predefined cancer-related genes was used to perform hybridization capture (**Table S1**). Enriched libraries were sequenced on Hiseq 4000 NGS platforms (Illumina) to target mean coverage depths of at least 100× for WBCs, 1000× for tissue- and bile pellet–derived DNA, and 5,000× for plasma- and bile supernatant–derived cfDNA.

### Data processing and analysis

Sequencing data were demultiplexed by bcl2fastq (v2.19) and analyzed by Trimmomatic to remove low-quality (quality<15) or N bases. Then, the data were aligned to the hg19 reference human genome with the Burrows–Wheeler Aligner (bwa-mem) and further processed using the Picard suite (available at https://broadinstitute.github.io/picard/) and the Genome Analysis Toolkit (GATK). Single-nucleotide variant (SNV) and small insertion and deletion (Indel) were called by VarScan2 and HaplotypeCaller/UnifiedGenotyper in GATK. Common single-nucleotide polymorphisms (SNPs) were removed using dbSNP and the 1,000 Genome data sets. Germline mutations were filtered out by comparing them to the oral swab controls. A mutation was called when the mutation allele frequency (MAF) cutoff was ≥ 0.5% for tissue and bile pellet samples, 0.2% for liquid biopsy samples, and a minimum of three unique mutant reads on different strands with good quality scores and manually inspected in Integrative Genomics Viewer Software (IGV, Broad Institute). Gene fusions were identified by FACTERA, and copy number variation (CNV) was analyzed with ADTEx. The mean percentage of genes with abnormal (log2 depth ratio > ± 0.2) copy numbers, weighted on 22 autosomal chromosomes, was defined as CIN score (CIS).

### Statistical analysis

Quantitative data are presented as median (range) or absolute number (percentage). Variables such as cfDNA concentration, MAF, number of genomic alterations, and CIS were compared using the Wilcoxon non-parametric test (Wilcoxon signed-rank test). The correlations of MAF between tumor tissue and the other sample types (i.e., plasma, bile supernatant or bile pellet) were calculated using the Spearman test. Two-tailed P-values <0.05 were considered statistically significant. All statistical analyses were performed with R 3.5.2.

## Results

### Clinicopathological data of patients with BTC

This study enrolled 13 patients with BTC, including seven men and six women. The median age was 63 years, and the median BMI was 23.84 kg/m^2^. Of the 13 patients, there were seven cases of gallbladder carcinoma, and six cases of cholangiocarcinoma (three cases of hilar cholangiocarcinoma, one of middle and upper bile duct carcinoma, and two cases of distal cholangiocarcinoma). Six patients were poorly differentiated, one was moderately poorly differentiated, and four were moderately differentiated. On the basis of the BTC staging of the seventh edition of the American Joint Committee on Cancer (AJCC), five patients were stage II, seven were stage III, and one was stage IV (**Table 1**).

**Table 1:** Clinicopathological characteristics in all patients enrolled in this study.

Characteristics	N (%)/Value
Age, median (range), years	63 (51–79)
Gender	
Female	6 (46.2%)
Male	7 (53.8%)
BMI, median (range), kg/m^2^	23.84 (14.92–28.95)
Hypertensive	7 (53.8%)
Diabetic	5 (38.5%)
Tumor type	
Cholangiocarcinoma Gallbladder carcinoma	6 (46.2%) 7 (53.8%)
Tumor location	
Gallbladder Hepatic hilar Intrahepatic bile duct Distal bile duct	7 (53.8%) 3 (23.1%) 1 (7.7%) 2 (15.4%)
Tumor differentiation	
Poorly differentiated Moderately poorly differentiated Moderately differentiated	6 (54.5%) 1 (9.1%) 4 (36.4%))
Bile collection method	
PTCD Intraoperatively	2 (15.4%) 11 (84.6%)
AJCC Stage	
I~II III~IV Tumor, median (range), cm Lymph node invasion	5 (38.5%) 8 (61.5%) 3 (2~6) 7 (63.6%)
ALB, median (range), g/L	39 (29.1–46.8)
TB, median (range), μmol/L	72.2 (8.3–421.6)
AFP, median (range), μg/L	1.5 (1.3–4.5)
CEA, median (range), ng/ml	11.0 (0.6–11.6)
CA19-9, median (range), U/ml	29.2 (1.2–1911.3)

BMI, body mass index; PTCD, percutaneous transhepatic cholangial drainage; ALB, albumin; TB, total bilirubin; AFP, alpha-fetoprotein; CEA, carcinoembryonic antigen; CA19-9, carbohydrate antigen 199.

### Comparison of mutation abundance/MAF

**Figure 1:**
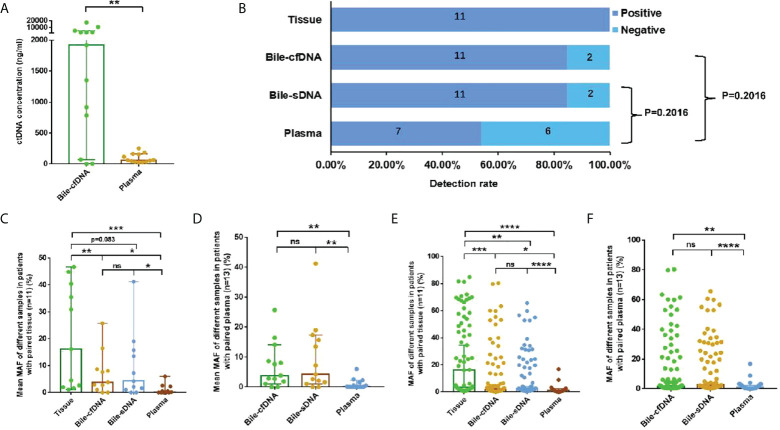
Detection rate and mutation allele frequency (MAF) of each sample type. **(A)** Comparison of cfDNA concentrations in 11 paired bile supernatants and plasma samples, median: 1,918 vs. 63.1 ng/ml, p = 0.0017. **(B)** Detection rate in tissue, bile supernatant, bile pellet, and plasma. **(C, D)** The comparison of the mean MAF of each sample in different sample types. **(E, F)** The comparison of median MAF of all mutations in different sample types. ns, not significant; *p-value < 0.05; **p-value < 0.01; ***p-value < 0.001; ****p-value < 0.0001; Wilcoxon signed-rank test.

Extracted cfDNA concentration was significantly higher in the bile supernatant than in plasma in the 11 cases with matched tumor tissues; median cfDNA of 1918 vs. 63.1 ng/ml, p = 0.0017 (**Figure 1A**). Mutations were detected in all tissue samples, and the positive detection rate was 100%. The positive rates for the bile supernatant and pellet were both 84.62% (11/13) and that for plasma was 53.85% (7/13) (**Figure 1B**). The mean MAF of each sample was significantly higher in the tumor tissue than in the bile supernatant, bile pellet, and plasma (16.13% vs. 3.84% vs. 4.22% vs. 0.16%) (**Figure 1C**). The mean MAF of each sample was significantly higher in the supernatant/pellet than in plasma (3.84% vs. 4.22% vs. 0.16%) (**Figure 1D**), with all p-values < 0.05 (Wilcoxon signed-rank test). For the 11 patients with tumor tissue samples, the median MAF of all mutations was 16.32%, 1.51%, 2.68%, and 1.20% in tissue, bile supernatant, bile pellet, and plasma, respectively. The median MAF of all mutations in the tissue was significantly higher than in the bile supernatant, bile pellet, and plasma (p < 0.05, Wilcoxon signed-rank test). Moreover, the median MAF of all mutations in both bile supernatant and pellet was significantly higher than in plasma (p < 0.05, Wilcoxon signed-rank test). However, the median MAF in bile supernatant and pellet was not significantly different (*p* > 0.05, Wilcoxon signed-rank test) (**Figure 1E**). In all 13 patients enrolled in this study, the median MAF of all mutations in both bile supernatant and pellet was also significantly higher than in plasma (p < 0.01, Wilcoxon signed-rank test); however, no difference was observed in the median MAF between bile supernatant and pellet (*p > 0.05*, Wilcoxon signed-rank test) (**Figure 1F**).

### Comparison of genomic alteration type and consistency

#### Genomic alterations detection in each sample type

**Figure 2:**
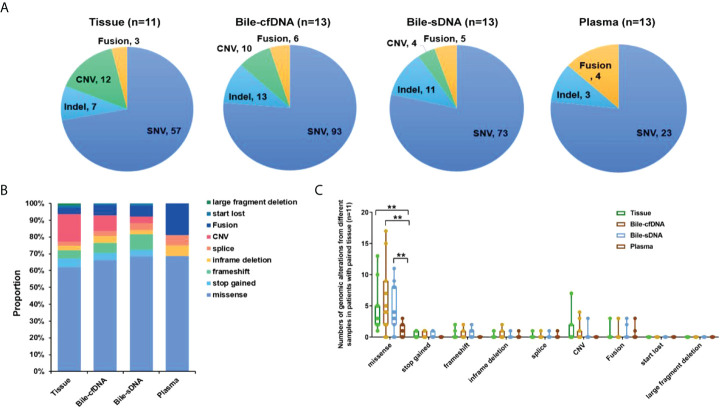
Distribution of genomic alterations types in different sample types. **(A)** Detected genomic alteration types in tissue, bile supernatant, bile pellet, or plasma. **(B)** The proportion of genomic alteration types in different samples in patients with paired tissues. **(C)** The number of various genomic alterations in different samples in patients with paired tissues. **p-value < 0.01; Wilcoxon signed-rank test.

A total of 79 genomic alterations in 57 genes were detected in 11 tumor tissue samples collected, including SNV (57, 72.15%), Indel (7, 8.86%), CNV (12, 15.19%), and fusion (3, 3.80%); 122 genomic alterations in 84 genes were detected in the bile supernatant of 13 bile-derived liquid biopsy samples collected, including SNV (93, 76.23%), Indel (13, 10.66%), CNV (10, 8.20%), and fusion (6, 4.92%). A total of 93 genomic alterations in 68 genes were detected in the pellet of the 13 bile-derived liquid biopsy samples. These included SNV (73, 78.47%), Indel (11, 11.83%), CNV (4, 4.30%), and fusion (5, 5.38%). A total of 30 mutations in 24 genes were detected in the 13 plasma samples, including SNV (23, 76.67%), Indel (3, 10.00%), and fusion (4, 13.33%) (**Figure 2A**).

#### Comparison of genomic alteration consistency

**Figure 3:**
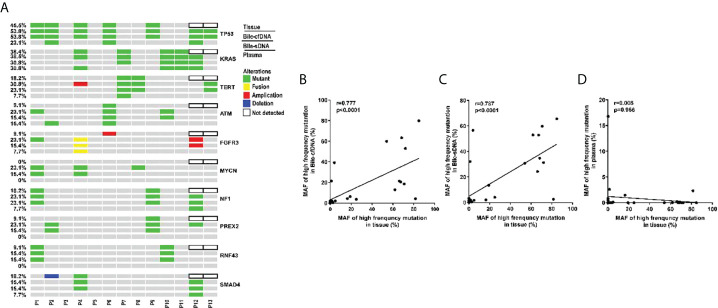
Gene genomic alteration profiles and the correlations of MAF between tumor tissue and the other sample types. **(A)** Profiles of recurrent gene genomic alterations in different sample types; MAF correlations between tissue and **(B)** bile supernatant, **(C)** bile pellet, and **(D)** plasma.

Genomic alteration types were predominantly missense in the tumor tissue and bile-derived liquid biopsy samples. Genomic alteration types in supernatant/pellet and corresponding tumor tissue samples were relatively consistent and were significantly different from those in plasma (p < 0.01). The number of missense mutations was significantly higher in supernatant/pellet than in plasma (p < 0.01) (**Figures 2B, C**). Of the 10 frequently mutated genes in BTC in this study, the consistency in genomic alterations between bile supernatant and tumor tissue was 90.0% (18/20), that between bile pellet and tumor tissue was 85.0% (17/20), and that between plasma and tumor tissue was 35.0% (7/20) (**Figure 3A**). For the 10 most frequently mutated genes, MAF in bile supernatant and pellet was positively correlated with that in tumor tissue (ρ < 0.0001, Spearman r = 0.777, and ρ < 0.0001, Spearman r = 0.787, respectively) (**Figures 3B, C**). There was no correction of MAF in plasma with that in tumor tissue (ρ = 0.966, Spearman r = 0.008) (**Figure 3D**). Bile supernatant and pellet showed higher MAF and consistency in 10 recurrently mutated genes compared with plasma.

### Potential clinical benefit of bile-derived liquid biopsy cfDNA genomic alterations

**Figure 4:**
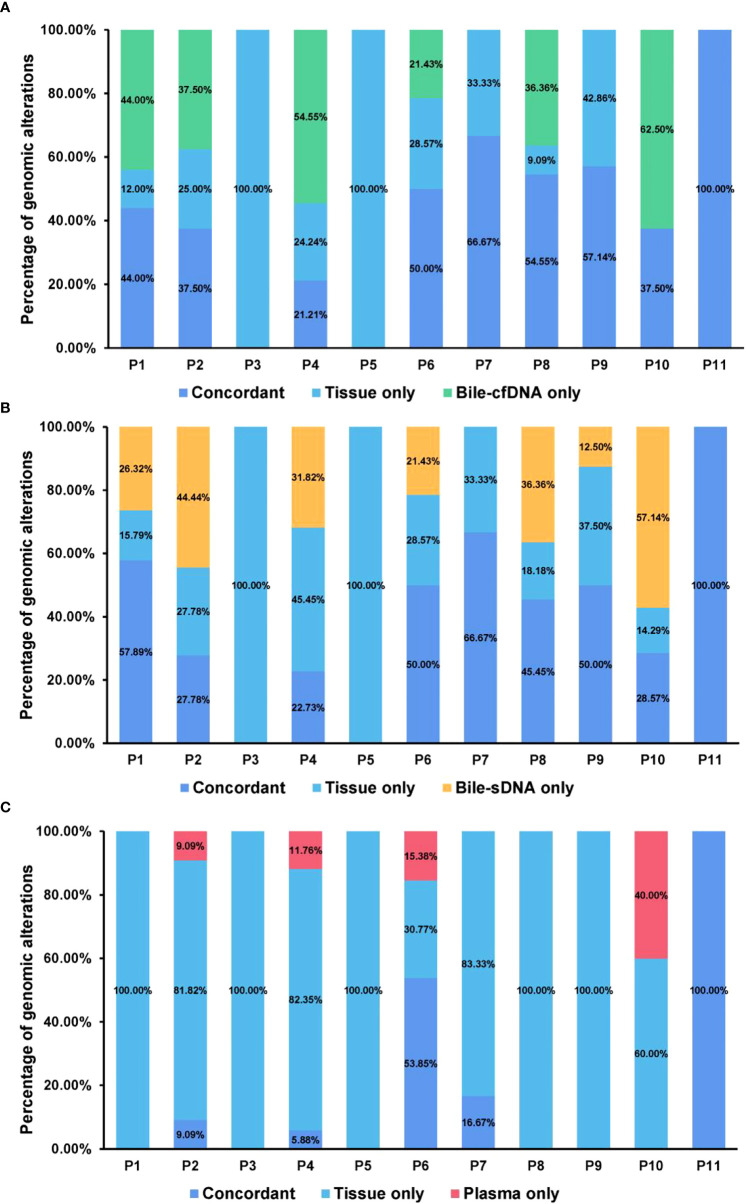
Genomic alteration consistency analysis between tumor tissue and the other sample types. Genomic alteration consistency between tissue and **(A)** bile supernatant, **(B)** bile pellet, and **(C)** plasma in 11 patients with BTC who provided tissue samples.

**Figure 5:**
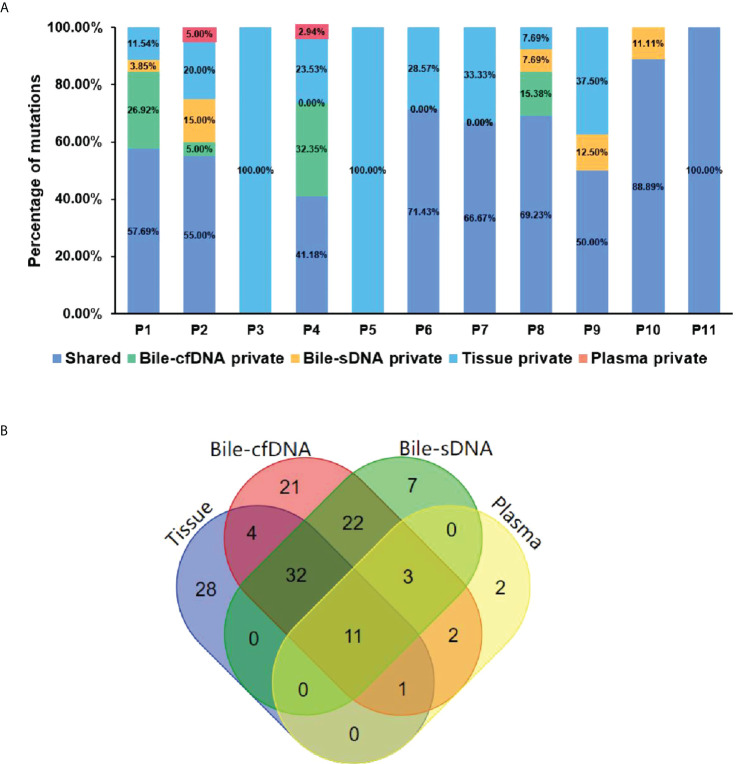
The shared or specific genomic alterations detected in different sample types. **(A)** The percentage of the shared or specific genomic alterations found in tissue, bile supernatant, bile pellet, and plasma. **(B)** Crossover diagram of genomic alteration number detected in different sample types.

**Figure 6:**
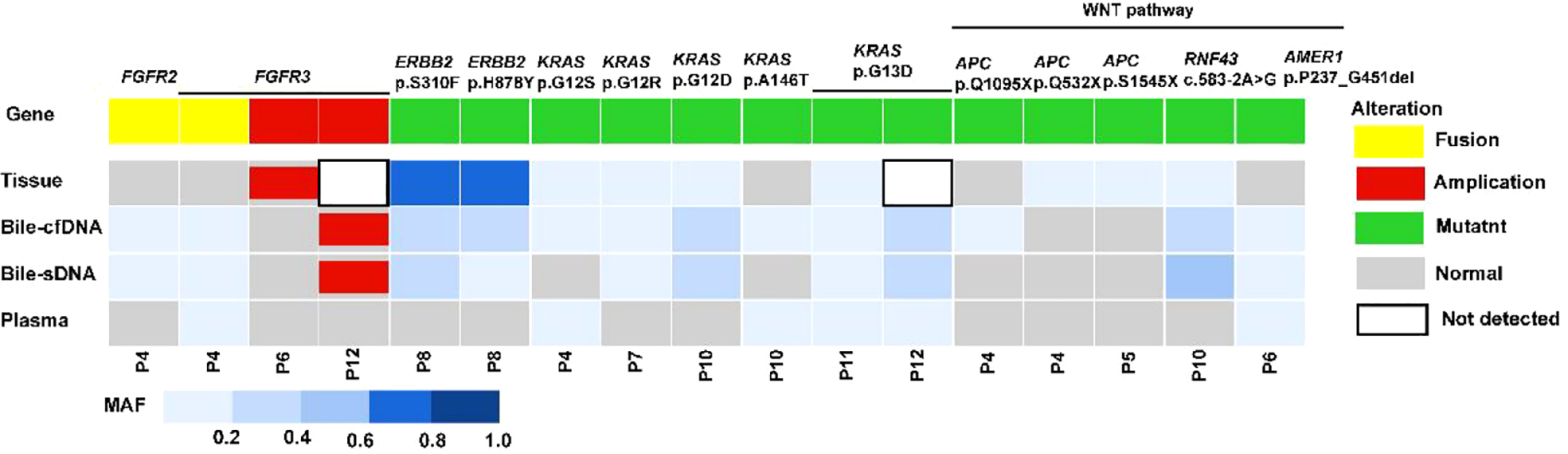
The potential targets of targeted therapy in BTC detected in patients in this study.

**Figure 7:**
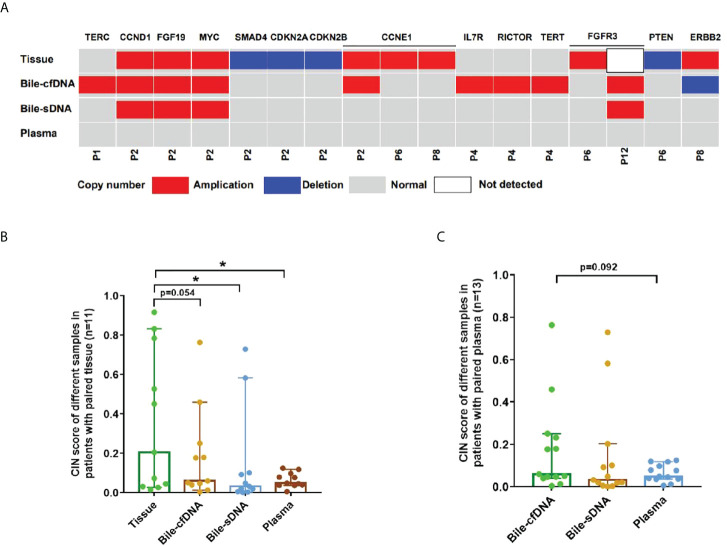
Copy number variation (CNV) and chromosome instability score (CIS) detected in this study. **(A)** CNV detected in each sample type. Comparison of CIS between tissue and bile supernatant, bile pellet, or plasma in **(B)** 11 patients who provided tissue samples and in **(C)** all patients enrolled. *p-value < 0.05; Wilcoxon signed-rank test.

On the basis of the results of the 11 patients with BTC who provided tumor tissue samples, there was more consistency in detected genomic alterations between bile supernatant/pellet and tumor tissue than between the tumor tissue and plasma (**Figures 4A-C**). Genomic alteration consistency between bile supernatant/pellet and tumor tissue was significantly higher than between the tissue and plasma (63.64% vs. 63.64% vs. 0%) (**Table 2**; **Figures 4A-C**). Furthermore, additional genomic alterations not found in tissue were detected in bile supernatant and pellet in more patients than in plasma (**Figures 4A–C**, **5A**). Genomic alteration consistency between supernatant and tumor tissue was 63.16% (48/76). Additional 48 genomic alterations were found in the bile supernatant that was absent in the tumor tissue (**Figure 5B**, **Table S2**). Consistency between bile pellet and tumor tissue was 56.58% (43/76). An extra 32 genomic alterations were detected in the bile pellet that was not detected in tumor tissue (**Figure 5B**, **Table S2**). Regarding potential targets of targeted therapy in BTC (*ERBB2*, *FGFR1*, *FGFR2*, *FGFR3*, and *KRAS* genes and WNT pathway), relevant genomic alterations were also detected in bile supernatant and pellet (**Figure 6**). For CNV and CIN detection, four additional CNVs from two patients that could not be detected in tumor tissues were detected in bile supernatant and pellet. The CIS of tumor tissue samples was significantly higher than that of plasma samples (p < 0.05), while there was no significant difference between tissue and bile supernatant (p = 0.054). The bile supernatant and pellet were more suitable for CNV detection than plasma (**Figure 7**).

**Table 2:** Detection sensitivity analysis of bile supernatant, bile pellet, and plasma for tumor tissue-determined genomic alterations.

		Bile-cfDNA	
		Positive	Negative
Bile-sDNA	Positive	45	0
	Negative	5	29
Plasma	Positive	12	0
	Negative	38	29

## Discussion

Traditional bile biopsy techniques include bile liquid-based cytology and detecting some proteins and bile components. Classic liquid-based cytology requires a larger bile volume, and repeated collection of 5 ml of bile until the tumor tissue sample can be obtained and the positive rate is relatively low (20%–40%) (6). In recent years, liquid biopsy has become an essential method for clinical diagnosis and treatment as a new diagnostic technique. Compared with other screening methods, liquid biopsy has the advantages of being noninvasive and easy, allowing reproducible acquisition of specimens. It can be used for prognosis evaluation, targeted treatment planning, and efficacy monitoring (7). In studies of several tumor types, such as non–small cell lung cancer and colorectal cancer, a liquid biopsy of blood specimens has been shown to partially replace tissue biopsy as a first-line test (7, 8). The above findings have only been substantiated in non–small cell lung and colorectal cancer, and the same has yet to be investigated in BTC.

In precision oncology, early diagnosis is crucial for treating and managing patients with BTC, and there is increasing emphasis on individualized treatment. Previous studies demonstrated the feasibility, accuracy, and sensitivity of bile-derived liquid biopsy as a candidate for genetic testing on NGS platforms. In a study of 10 patients with BTC, bile and tumor were examined using NGS in 150 tumor-associated genes; the results showed that the SNV/Indel of Bile-cfDNA had high sensitivity (94.7%) and specificity (99.9%) and that the CNV has a sensitivity of 75.0% and a specificity of 98.9% (9). In another study, bile samples from 21 patients with pancreatic ductal adenocarcinoma or BTC were detected by NGS, and 96.2% of tumor-determined mutations were found. In contrast, only 31.6% of tumor-determined mutations were detected in plasma-derived cfDNA (10). Previous studies almost focused on the bile supernatant and the mutation consistency between bile supernatant and tumor tissue. Our study not only explored the assay performance of bile supernatant on genetic testing but also investigated the performance of bile pellet.

For patients with BTC, the effect of using bile for NGS is far superior to plasma. On the one hand, in obstructive jaundice caused by tumors, biliary epithelial cells and cancer cells are detectable in bile. The concentration of ctDNA in bile can be very high (11). This was also demonstrated in this study, with cfDNA concentrations in the bile supernatant being significantly higher than those in plasma (1918 vs. 63.1 ng/ml, p = 0.0017). However, it is imperative to determine the source of ctDNA. If NGS is performed directly on plasma, since some organs and tissues in the body may release ctDNA into the bloodstream, then we cannot definitively determine the source of ctDNA. The situation is different for bile. In previous studies, *TP53*, *ERBB2*, and *KRAS*, genes closely related to gallbladder cancer, were detected not only in the tumor tissue but also in bile. Furthermore, the two had a high degree of consistency (12). In this study, we also found that, among the 10 high-frequency genomic alterations in BTC, the consistency between bile supernatant and tumor tissue was 90% and that between the pellet and the tumor tissue was 85%. MAF in the supernatant was positively correlated with that in the tumor tissue (ρ < 0.0001, Spearman r = 0.777), and MAF in the pellet was also positively correlated with that in the tumor tissue (ρ < 0.0001, r = 0.787). This was not the case for plasma (ρ = 0.966, Spearman r = 0.008), which further demonstrated that bile ctDNA was derived from BTC. As such, bile is a suitable source of cfDNA for BTC compared with plasma.

For patients with BTC, early surgical resection is the only curative option; however, only 20%–40% of patients have the opportunity to undergo resection, and many experience postoperative recurrence (13). Postoperative cisplatin + gemcitabine adjuvant therapy in combination with 5-fluorouracil + oxaliplatin first-line therapy may improve patients’ overall survival; however, the efficacy is limited. The associated postoperative mean survival is less than 1 year (14, 15). A recent study also demonstrated the effectiveness of immunotherapy in patients with BTC with high expression of programmed death ligand-1 and programmed death-1 (16). Improving patient survival and prognosis is particularly difficult in clinical practice for patients with advanced unresectable BTC. In this study, we found that many patients with BTC had a high mutation frequency of oncogenes such as *KRAS* (i.e., p.G12S, p.G12R, p.G12D, and p.G13D) and *TP53* (i.e., p. R282W, p.P222Sfs*26, p.R283_E285delinsHTK, and c.560-1G>C) in both bile and tumor tissues. We deduce that bile can accurately reflect the physiological status of the tumor tissue. We also detected tumor tissue-determined genomic alterations in bile, such as *FGFR3* and *MYCN.* This further supports consistency between the bile and tumor tissue samples. In addition, bile could be used to detect genomic alterations in BTC that are cancer-targeted therapy-related candidates, such as the *ERBB2*, *FGFR1*, *FGFR2*, *FGFR3*, *KRAS*, and WNT pathways. This may be valuable in targeted BTC treatment.

CIN is one of the most common features of tumor genes. CIN not only plays a role in the oncogenesis, development, and spread of tumors but is also considered a common player in tumor resistance to therapy. CIN underpins much of the intratumor heterogeneity, driving phenotypic adaptation during tumor evolution and ultimately leading to poor clinical outcomes and accelerating resistance to treatment in various tumors (17, 18). In treating some tumors, such as gastric and breast cancers, CIN also plays a role in monitoring the efficacy of treatment and patient prognosis (19, 20). In this study, we observed four additional CNVs in bile supernatant that were not detected in tumor tissues. No CNVs detected in tissue were observed in plasma, and the CIN was significantly higher in the tumor tissue than in plasma (p < 0.05). However, there was no significant difference in the case of bile supernatant compared with tissue. Therefore, we reason that bile is more suitable for detecting CNV and CIN than plasma for patients with BTC. Consequently, it can be used to monitor treatment efficacy and evaluate prognosis.

There are some limitations in the present study. First, the most significant limitation of this study was the very small sample size. Furthermore, whether the efficiency in genetic testing of bile is different between cholangiocarcinoma and gallbladder cancer was still unknown. Therefore, more studies with larger sample sizes are needed to confirm the findings of this study. Second, we examined the potential therapeutic targets detected by bile in this study. No clinical treatments have been carried out yet. The predictive value of potential therapeutic targets detected in bile for clinical efficacy is unknown; future research in this area is needed.

## Conclusion

NGS could detect cfDNA in bile, and the genomic alterations detected in bile were highly consistent with those detected in tumor tissue. Bile is superior to plasma for NGS in patients with BTC and can be used to identify potential targets to guide targeted clinical therapy. It may be feasible to replace tumor tissue with bile cfDNA in genetic sequencing, and liquid biopsy of bile could provide a more reasonable, personalized, and well-directed option for the treatment and clinical management of patients with BTC.

## Data availability statement

The original contributions presented in the study are included in the article/**Supplementary Material**. Further inquiries can be directed to the corresponding author.

## Ethics statement

The studies involving human participants were reviewed and approved by Department of Hepato-Pancreato-Biliary Surgery, Ningbo Medical Center Lihuili Hospital, The affiliated hospital of Ningbo University. The patients/participants provided their written informed consent to participate in this study.

## Author contributions

ZL wrote the manuscript. YL and JF contributed to the literature search. ZL, JY, and JM revised the manuscript. JY performed the data analysis and created the figures. CL and JH designed the research. All authors contributed to the study and approved the submitted version.

## Funding

This work was supported by Ningbo Medical and Health Brand Discipline (PPXK2018-03) and Ningbo Public Welfare Project(2021S187).

## Acknowledgments

We would like to thank the patients and family members who gave their consent to present the data in this study.

## Conflict of interest

JY is employee of Nanjing Geneseeq Technology Inc.

The remaining authors declare that the research was conducted in the absence of any commercial or financial relationships that could be construed as a potential conflict of interest.

## Publisher’s note

All claims expressed in this article are solely those of the authors and do not necessarily represent those of their affiliated organizations, or those of the publisher, the editors and the reviewers. Any product that may be evaluated in this article, or claim that may be made by its manufacturer, is not guaranteed or endorsed by the publisher.
