# Supplementary material for: Bile is a reliable and valuable source to study cfDNA in biliary tract cancers
Source: Front Oncol. 2022 Aug 26;12:961939. doi: 10.3389/fonc.2022.961939 (PMC9459008; doi:10.3389/fonc.2022.961939)
Supplement: Supplementary file 1 [file Table_1.docx]

Table S1. The list of target genes detected in NGS of this study

| ABCB1(MDR1) | ABCC2(MRP2) | ADH1B | AIP | AKT1 | AKT2 |
| --- | --- | --- | --- | --- | --- |
| AKT3 | ALDH2 | ALK | AMER1(FAM123B) | APC | AR |
| ARAF | ARID1A | ARID1B | ARID2 | ARID5B | ASCL4 |
| ASXL1 | ATF1 | ATIC | ATM | ATR | ATRX |
| AURKA | AURKB | AXIN2 | AXL | B2M | BAD |
| ADGRB3(BAI3) | BAK1 | BAP1 | BARD1 | BAX | BCL2 |
| BCL2L11(BIM) | BCR | BIRC3 | BLM | BMPR1A | BRAF |
| BRCA1 | BRCA2 | BRD4 | BRIP1 | BTG2 | BTK |
| BUB1B | EMSY(c11orf30) | CASP8 | CBL | CBLB | CCND1 |
| CCNE1 | CD274(PD-L1) | CD74 | CDA | CDC73 | CDH1 |
| CDK10 | CDK12 | CDK4 | CDK6 | CDK8 | CDKN1A |
| CDKN1B | CDKN1C | CDKN2A | CDKN2B | CDKN2C | CEBPA |
| CEP57 | CHD4 | CHD8 | CHEK1 | CHEK2 | CREBBP |
| CRKL | CSF1R | CTCF | CTLA4 | CTNNB1 | CUL3 |
| CUX1 | CXCR4 | CYLD | CYP19A1 | CYP2A13 | CYP2A6 |
| CYP2A7 | CYP2B6 | CYP2C19 | CYP2C9 | CYP2D6 | CYP3A4 |
| CYP3A5 | CYSLTR2 | DAXX | DDR2 | DENND1A | DHFR |
| DICER1 | DLL3 | DNMT3A | DOT1L | DPYD | DTL(CDT2) |
| DUSP2 | EGFR | EIF1AX | EP300 | EPAS1 | EPCAM |
| EPHA2 | EPHA3 | EPHA5 | ERBB2(HER2) | ERBIN(ERBB2IP) | ERBB3 |
| ERBB4 | ERCC1 | ERCC2 | ERCC3 | ERCC4 | ERCC5 |
| ESR1 | ETV1 | ETV4 | ETV5 | ETV6 | EWSR1 |
| EXT1 | EXT2 | EZH2 | EZR | FANCA | FANCC |
| FANCD2 | FANCE | FANCF | FANCG | FANCI | FANCL |
| FANCM | FAT1 | FBXW7 | FGF19 | FGFR1 | FGFR2 |
| FGFR3 | FGFR4 | FH | FLCN | FLT1(VEGFR1) | FLT3 |
| FLT4 | FOXA1 | FOXL2 | FOXP1 | FRG1 | GATA1 |
| GATA2 | GATA3 | GATA4 | GATA6 | GNA11 | GNAQ |
| GNAS | GRIN2A | GRM3 | GRM8 | GSTM1 | GSTM4 |
| GSTP1 | GSTT1 | HDAC2 | HDAC9 | HGF | HLA-A |
| HNF1A | HNF1B | HRAS | IDH1 | IDH2 | IFNA6 |
| IFNB1 | IFNE | IFNG | IFNGR1 | IFNGR2 | IGF1R |
| IGF2 | IKBKE | IKZF1 | IL7R | INPP4B | IRF2 |
| JAK1 | JAK2 | JAK3 | JARID2 | JUN | KDM5A |
| KDR(VEGFR2) | KEAP1 | KIF1B | KIT | KITLG | KLLN |
| KMT2A(MLL) | KMT2B | KMT2C | KMT2D(MLL2) | KRAS | LHCGR |
| LMO1 | LRP1B | LYN | LZTR1 | MAP2K1(MEK1) | MAP2K2(MEK2) |
| MAP2K4 | MAP3K1 | MAP3K4 | MAX | MCL1 | MDM2 |
| MDM4 | MECOM | MED12 | MEF2B | MEN1 | MET |
| MGMT | MITF | MLH1 | MLH3 | MLLT1 | MLLT3 |
| AFDN(MLLT4) | MPL | MRE11(MRE11A) | MSH2 | MSH6 | MTHFR |
| MTOR | MUTYH | MYC | MYCL(MYCL1) | MYCN | MYD88 |
| MYH9 | NAT1 | NBN | NCOR1 | NF1 | NF2 |
| NFE2L2 | NFKBIA | NKX2-1 | NOTCH1 | NOTCH2 | NOTCH3 |
| NPM1 | NQO1 | NRAS | NRG1 | NSD1 | NTRK1 |
| NTRK2 | NTRK3 | NUTM1 | PAK3 | PALB2 | PALLD |
| PRKN(PARK2) | PARP1 | PARP2 | PAX5 | PBRM1 | PDCD1(PD1) |
| PDCD1LG2(PD-L2) | PDE11A | PDGFRA | PDGFRB | PDK1 | PGR |
| PHOX2B | PIK3C3 | PIK3CA | PIK3CD | PIK3R1 | PIK3R2 |
| PKHD1 | PLAG1 | PLCB4 | PLK1 | PMS1 | PMS2 |
| POLD1 | POLD3 | POLE | POLH | POT1 | PPARD |
| PPP2R1A | PRDM1 | PREX2 | PRF1 | PRKACA | PRKAR1A |
| PRKCI | PRKDC | PRSS1 | PRSS3 | PTCH1 | PTEN |
| PTK2 | PTPN11 | PTPN13 | QKI | RAC1 | RAC3 |
| RAD50 | RAD51 | RAD51B | RAD51C | RAD51D | RAD54L |
| RAF1 | RARA | RARG | RASGEF1A | RB1 | RECQL4 |
| RELN | RET | RHOA | RICTOR | RNF43 | ROS1 |
| RPTOR | RRM1 | RUNX1 | RUNX1T1 | SBDS | SDC4 |
| SDHA | SDHB | SDHC | SDHD | SEPTIN9(SEPT9) | SETBP1 |
| SETD2 | SF3B1 | SGK1 | SKP2 | SLC34A2 | SLC3A2 |
| SMAD2 | SMAD3 | SMAD4 | SMAD7 | SMARCA4 | SMARCB1 |
| SMO | SOCS1 | SOS1 | SOX2 | SPOP | SPRED1 |
| SPRY4 | SRC | SRSF2 | SRY | STAG2 | STAT3 |
| STK11 | STMN1 | SUFU | TACC3 | TAP1 | TAP2 |
| TEK | TEKT4 | TERC | TERT | TET2 | TGFBR2 |
| THADA | TMEM127 | TMPRSS2 | TNFAIP3 | TNFRSF11A | TNFRSF14 |
| TNFRSF19 | TNFSF11 | TOP1 | TOP2A | TP53 | TP63 |
| TPMT | TSC1 | TSC2 | TSHR | TTF1 | TUBB3 |
| TYMS | U2AF1 | UGT1A1 | VAMP2 | VEGFA | VHL |
| WAS | CCN6(WISP3) | WRN | WT1 | XPA | XPC |
| XRCC1 | XRCC2 | YAP1 | ZNF217 | ZNF703 |  |
